# Genomic Characterization Provides New Insights for Detailed Phage- Resistant Mechanism for *Brucella abortus*

**DOI:** 10.3389/fmicb.2019.00917

**Published:** 2019-05-03

**Authors:** Xu-ming Li, Yao-xia Kang, Liang Lin, En-Hou Jia, Dong-Ri Piao, Hai Jiang, Cui-Cai Zhang, Jin He, Yung-Fu Chang, Xiao-Kui Guo, YongZhang Zhu

**Affiliations:** ^1^Baotou Municipal Center for Disease Control and Prevention, Baotou, China; ^2^Stake Key Laboratory of Agricultural Microbiology, College of Life Science and Technology, Huazhong Agricultural University, Wuhan, China; ^3^State Key Laboratory for Infectious Disease Prevention and Control, Collaborative Innovation Center for Diagnosis and Treatment of Infectious Diseases, National Institute for Communicable Disease Control and Prevention, Beijing, China; ^4^Collaborative Innovation Centre for Diagnosis and Treatment of Infectious Diseases, Zhejiang University, Hangzhou, China; ^5^Department of Immunology and Microbiology, Institutes of Medical Sciences, Shanghai Jiao Tong University School of Medicine, Shanghai, China; ^6^Department of Population Medicine and Diagnostic Sciences, College of Veterinary Medicine, Cornell University, Ithaca, NY, United States

**Keywords:** *Brucella abortus*, phage resistance, comparative genomics, genome typing, phylogenetic analysis

## Abstract

As the causative agent of cattle brucellosis, *Brucella abortus* commonly exhibits smooth phenotype (by virtue of colony morphology) that is characteristically sensitive to specific *Brucella* phages, playing until recently a major role in taxonomical classification of the *Brucella* species by the phage typing approach. We previously reported the discrepancy between traditional phenotypic typing and MLVA results of a smooth phage-resistant (SPR) strain Bab8416 isolated from a 45-year-old custodial worker with brucellosis in a cattle farm. Here, we performed whole genome sequencing and further obtained a complete genome sequence of strain Bab8416 by a combination of multiple NGS technologies and routine PCR sequencing. The detailed genetic differences between *B. abortus* SPR Bab8416 and large smooth phage-sensitive (SPS) strains were investigated in a comprehensively comparative genomic study. The large indels between *B. abortus* SPS strains and Bab8416 showed possible divergence between two evolutionary branches at a far phylogenetic node. Compared to *B. abortus* SPS strain 9-941 (Bab9-941), the specific re-arrangement event in Bab8416 displaying a closer linear relationship with *B. melitensis* 16M than other *B. abortus* strains resulted in the truncation of c-di-GMP synthesis, and 3 c-di-GMP-metabolizing genes, were present in Bab8416 and *B. melitensis* 16M, but absent in Bab9-941 and other *B. abortus* strains, indicating potential SPR-associated key determinants and novel molecular mechanisms. Moreover, despite almost completely intact smooth LPS related genes, only one mutated OmpA family protein of Bab8416, functionally related to flagellar and efflux pump, was newly identified. Several point mutations were identified to be Bab8416 specific while a majority of them were verified to be *B. abortus* ST2 characteristic. In conclusion, our study therefore identifies new SPR-associated factors that could play a role in refining and updating *Brucella* taxonomic schemes and provides resources for further detailed analysis of mechanism for *Brucella* phage resistance.

## Introduction

Brucellosis is one of the most serious zoonotic infectious diseases worldwide, and is caused by pathogenic species of *Brucella* genus. Up to now, 12 species were defined into the genus *Brucella* (Godfroid et al., 2013). Six of them, including *B. melitensis*, *B. abortus, B. suis*, *B. canis*, *B. ovis*, and *B. neotomae*, belong to the “classical” or “traditional” *Brucella* species^1^. Generally, all *Brucella* species with nucleotide similarities > 90% are genetically closely related (Al Dahouk et al., 2010).

Traditional *Brucella* typing is primarily based on different phenotypic characteristics (Garcia et al., 1988; Jahans et al., 1997; Moreno et al., 2002; Sanogo et al., 2013), including colony morphology, CO_2_ requirement, H_2_S production, substrate utilization, growth on serum dextrose agar dye plate, agglutination with monospecific sera, *Brucella* phage lysis profiles at routine test dilution (RTD) and host preference (Jones et al., 1968; Morris et al., 1973; Rigby et al., 1989). The three major species in terms of disease and economic impact for man, *B. melitensis*, *B. abortus* and *B. suis* are further subdivided into multiple biovars (*bv*) based on a range of phenotypic and serological characteristics. For example, *B. abortus* is subdivided into *bv* 1–6 and 9 (Pappas et al., 2006). Furthermore, despite the close genetic relationship of several genetic loci (e.g., 16S rRNA, 98.7%) and a biochemical profile similar to *Ochrobactrum* spp., several non-classical *Brucella* species like *B. microti* and *B. inopinata* are often easily misidentified using traditional biochemical typing methods (Scholz et al., 2008a, b). Among these routine phenotypic characterizations, *B. abortus* with smooth Lipopolysaccharide (LPS) was identified to be sensitive to *Brucella* phages like Berkeley2 (BK2), Tbilisi (Tb), Weybridge (Wb), and Izatnagar (Iz) (FAO/WHO, 1986). This useful test is significant for differentiating *B. abortus* from other *Brucella* species (Jones et al., 1968; Morris et al., 1973).

Since SPR *B. abortus* was initially reported in Corbel and Morris (1974), there have been few studies on this distinct phenotype over the last four decades. The susceptibility of smooth *B. abortus* strains to lysis by *Brucella* phages is commonly used to type various *Brucella* species. We have recently reported the identification of the first SPR *B. abortus* strain Bab8416 from a brucellosis patient in China (Kang et al., 2015). The phage activity of Bab8416 is similar to that of *B. melitensis bv* 1 strain 16M and showed special biochemical characteristics distinct from that of all *B. abortus* biovars. It was not lysed by Tb, Iz, and Wb phage in 1 × RTD and 10^4^ × RTD, but lysed by BK2 phage in 1 × RTD and 10^2^ × RTD. Due to the unusual discrepancy between phenotypic profiles, Bab8416 could not be precisely classified to any of the existing *B. abortus* biovars. In this study, we completed the genome sequence of Bab8416 through a combination of next-generation sequencing (NGS) and common PCR-based gap closure and investigated genomic differences between Bab8416 and other *Brucella* strains for gene association in corresponding biochemical or physiological profiles.

## Materials and Methods

### Ethics Statement

This study and the protocol were carried out in accordance with the recommendations of ethics committee of the local disease control and Prevention Research Center of the Inner Mongolia Autonomous Region and Baotou City. The patient gave written informed consent for participation in this study and publication of his identifiable information, in accordance with the Declaration of Helsinki. The detailed information of strain Bab8416 referred to our previous study (Kang et al., 2015).

### Genome Sequencing, Assembly and Annotation

Using 454 GS-FLX system, a total of 190,817 reads were obtained with the average length of 566 bp. Twenty-two contigs with lengths more than 500 bp and average coverage of 33.2X were obtained by Newbler using default parameters. Using the genome of *B. abortus* 9-941 as a reference, the order of the contigs was sorted and gap closure using common PCR was performed with ContigsScape (Tang et al., 2013). To fix the homopolymer sequencing errors systemically caused by 454 GS-FLX sequencing system, another 180 bp Paired End (PE) library was constructed and sequenced by the Illumina Hiseq 2000 system. Genome sequencing results were refined by short reads using Pilon with default parameters (Walker et al., 2014). The coding genes were predicted by Prodigal (Hyatt et al., 2010) and these genes were annotated by BLAST against NCBI non-redundant (NR), COG, KEGG, TrEMBL, Swissprot databases with e value cutoff of 1e-5 and GO terms assigned to the annotated genes using BLAST2GO pipeline (Conesa et al., 2005). The tRNAs were detected by tRNAscan-SE (v1.23) (Schattner et al., 2005) and rRNAs were identified by blasting homologous rRNA sequences against the Bab8416 genome.

### Whole Genome Collinear Analysis

Firstly, oriC site was identified in both references and Bab8416 genome using Ori-Finder 2 and was set to be the first base of Bab8416 genome (Luo et al., 2014). Then, whole genome sequence alignments between these two genomes were processed by MUMmer 3.23 package (Kurtz et al., 2004).

### *Brucella* MLVA Typing and MLST Typing

Multiple-locus variable number tandem repeat analysis (MLVA) assay was employed and the markers were obtained by PCR (Jiang et al., 2013b). The MLVA markers of Bab8416 were compared to the MLVA database^2^. The multilocus sequence typing (MLST) schemes of *Brucella* species using 9 conserved housekeeping genes were performed as previously described (Whatmore et al., 2007).

### SNP Calling

All the draft genomes were linked to be two pseudo chromosomes by taking *B. abortus* 9–941 genome as a reference and the sequences were gaped with ‘NNNNN.’ The SNPs were firstly identified by Mauve (Darling et al., 2010) using the genome sequences in this study and after “N” removed, the remaining SNP were finally exported for further analysis.

### Gene Family Identification and Phylogenetic Analysis

Thirty-nine available *Brucella* reference genomes were utilized to perform comparative genomic and phylogenetic analyses, including all known *Brucella* species and all of seven biovars of *B. abortus*. Three strains with lower contig numbers and high coverage in each biovar of *B. abortus* were selected. All genes of the selected strains were ortholog clustered by PGAP (Zhao et al., 2012), a pipeline for pan-genome analysis, and genes with both coverage and identity higher than 90% were considered to be the same ortholog cluster. Hence, a total of 2,014 single copy gene families were identified and a super gene was constructed for phylogenetic analysis by combining all sequences of these genes into one ortholog cluster. A maximum likelihood phylogenetic tree was constructed by Phyml 3.0 (Guindon et al., 2010) using HKY85 nucleotide substitution model with a bootstrap value of 1000. In addition, in order to investigate the regions of differences (RD) from pan-genome analysis, we further added 200 *B. melitensis* genomes and 197 *B. abortus* genomes for detailed screening and characterization by using BLASTN program.

### Virulence Factor Screening

We downloaded all the virulence factor from Virulence Factors Database (VFDB) (Chen et al., 2005), and we aligned all the protein sequences of the strain Bab8416 to the VFDB using BLASTP program available at NCBI server (ncbi-blast-2.7.1+) with both coverage and identity higher than 80%.

### Data Access

The genome sequence and annotations were submitted to GenBank database with accession number CP008774–CP008775. All the reference genomes used in this paper were obtained from PATRIC (Wattam et al., 2017).

## Results and Discussion

### Genome Features

**FIGURE 1:**
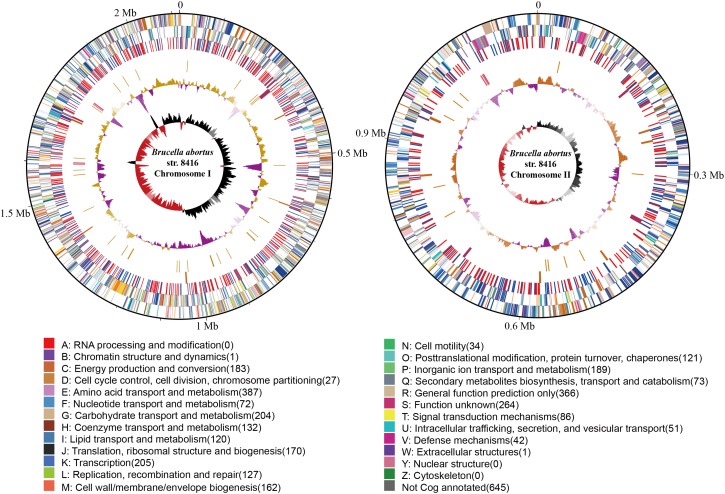
The atlas of *Brucella abortus* SPR strain Bab8416 genome. The outer black circle shows coordinates. Moving inward, the next two circles show forward and reverse strand CDS, respectively, with colors representing the functional classification, the next circle shows Bab8416 specific synonymous (red) and non-synonymous (blue) SNP, followed by the Bab8416 unique insertions (dark orange) and deletions (maroon), then is the tRNA (orange) and rRNA (deep pink). The final two are GC-content and GC-skew by using a 10-kb window and overlap at 1,000bp.

**FIGURE 2:**
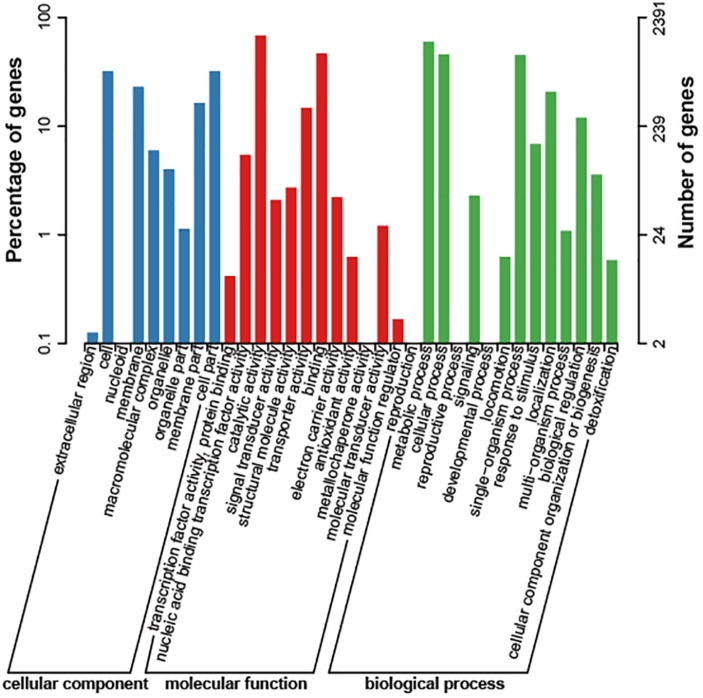
Cog class of *Brucella abortus* 8416 function genes. The result was export with *e*-value of 1e-5.

The genome size of strain Bab8416 is 3.2 Mb, and it consists of two circular chromosomes: a large chromosome of 2,116,946 bp and a smaller one of 1,156,123 bp. The average GC content of two chromosomes was 57.22% (Crasta et al., 2008; Tsolis et al., 2009). A total of 3,295 Coding DNA sequences (CDSs) have been computationally predicted. The summarized message of Bab8416 genome is showing in Figure 1. The average length of CDS was 856 bp and 2,272 CDSs (68.95%) were assigned definite biological function as well as 1,023 (31.05%) are hypothetical proteins. Figure 2 is showing GO function class of the annotated genes.

### Inconsistent Phenotypic and Molecular Typing Results

**FIGURE 3:**
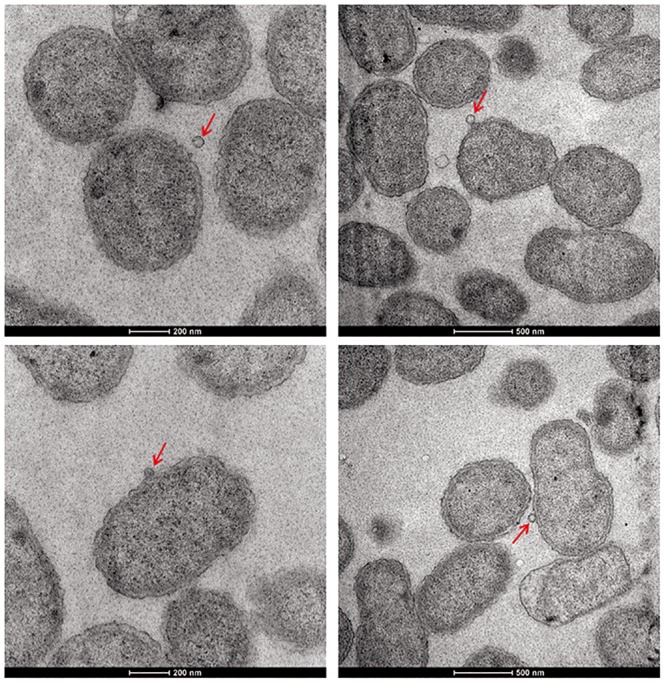
The electron microscope photo of interaction between *Brucella* phage and *Brucella abortus* SPR strain Bab8416. *Brucella* phages Tb phages were found successfully to adhere on the surface of *B. abortus* strain **Bab8416** but failed to lyse the strain. Red arrows are showing the dissociative Tb or Tb binding to strain 8416.

**FIGURE 4:**
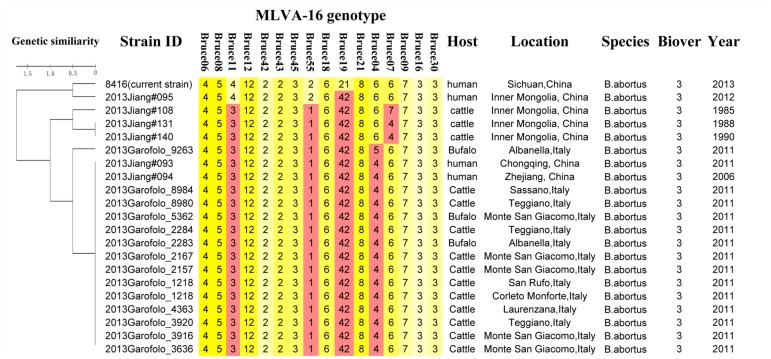
MLVA typing results of *B. abortus* strain Bab8416.

Except for resistance to phage Iz, Tb, and Wb shown in Table 1, the physiological and biochemical profiles of strain Bab8416 was more closely related to smooth *B. abortus bv* 9 (Morris et al., 1973). In addition, electron microscopy was used to investigate phage Tb/Bab8416 interaction (Figure 3); absorption but no lysis of host bacteria was observed. Here, we performed additional MLVA typing (Le Fleche et al., 2006; Al Dahouk et al., 2007; Van Belkum, 2007; Valdezate et al., 2009). While no 100% match could be found in MLVA database, the top 20 matches consistently with *B. abortus bv.* 3 (Figure 4).

**TABLE 1:** Physiological and biochemical typing details of *B. abortus* Bab8416 compared with other standard strains.

					Monospecific						
No	Growth characteristics				sera		Phages at RTD				Interpretation
	CO_2_ requirement	H_2_S production	TH	BF	A	M	Tb	Wb	Iz	BK2	
1	−	+	+	+	−	+	−	−	−	+	*B. abortus* 8416
2	±	+	+	+	+	−	+	+	+	+	*B. abortus* 3a
3	−	+	+	+	−	+	+	+	+	+	*B. abortus 9*
4	−	−	+	+	−	+	−	−	+	+	*B. melitensis* 16M

*TH, Thionin at 20 μg/ml (1/50,000); BF, Basic fuchsin at 20 μg/ml (1/50,000), Phages: Tb, Tbilisi; Wb, weybridge; BK2, Berkeley type 2; Fi, Firenze; RTD, Routine test dilution; +, positive; −, negative.*

Without coincident results in both traditional phenotyping and modern MLVA genotyping, we further employed MLST method (Whatmore et al., 2007). Twenty-seven *Brucella* sequence types (STs) were initially identified and more STs have been found (Whatmore et al., 2016). Bab8416 was identified as an ST2 in this study.

### Phylogenetic Analysis

**FIGURE 5:**
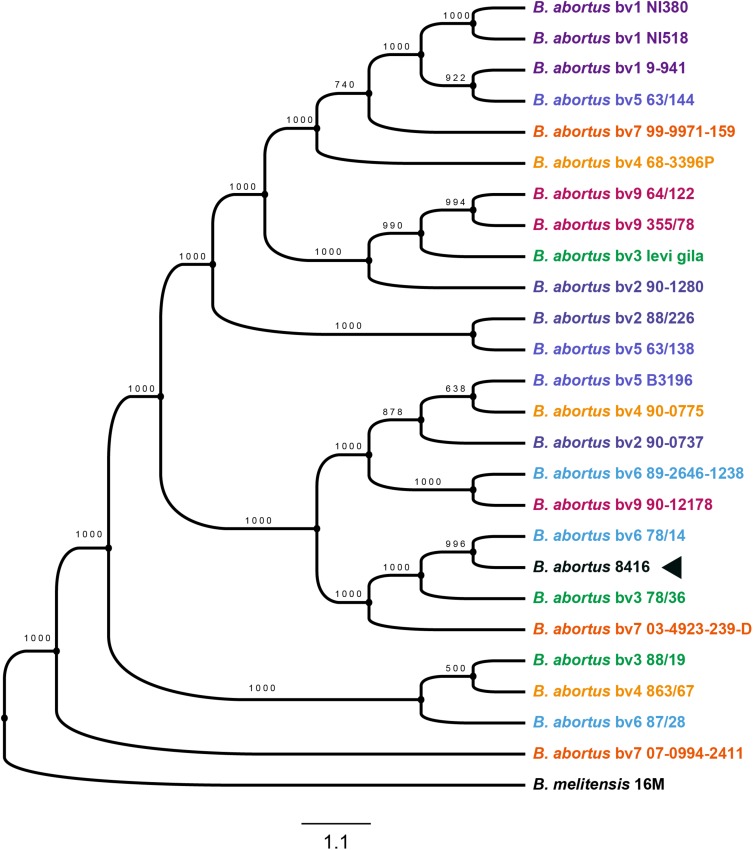
Phylogenetic tree of *Brucellae.* The phylogenetic tree was based on the 601 core genes of strains used in this analysis and it was constructed by using the maximum likelihood method with bootstrap value 1000. Black arrow is showing the phylogenetic cluster of *B. abortus* strain 8416.

Determining the evolutionary context of Bab8416 is essential for a detailed comparative genomic analysis and to account for the inconformity of the former two typing results from different strains and isolates of *Brucella* (Crasta et al., 2008). A total of 2,014 single copy genes were identified within 25 *B. abortus* strains with three strains in each biovar and *B. melitensis* str. 16M as one outgroup being used to build a maximum likelihood phylogenetic tree (Figure 5). Many strains within the same biovar are not closely genetically related; conversely, several strains in different biovars have been shown to be closely related. This finding indicates that traditional physiological and biochemical typing designations of biovars within *B. abortus* do not reflect genetic linkage patterns.

### Comparative Genomics

As draft genomes often generate low resolution results in studies measuring genetic variation, we conducted a comparative genomic analysis using complete genomes as previously described (Ricker et al., 2012; Zhang et al., 2012). *B. abortus* strain 9-941 (Bab9-941) was a typical SPS strain with the complete genome published. Here, we chose Bab9-941 as a reference for comparative genomic analysis and the genome features of *B. abortus* used and were listed in Table 2.

**TABLE 2:** Genome features of these strains used in comparative analysis.

Strains	Genome status	Biovar	Contig	CDS
*B. abortus* 8416	Complete	–	2	3295
*B. abortus* 9-941	Complete	1	2	3085
*B. abortus* 104M	WGS	–	92	3303
*B. abortus* 2308-A	WGS	1	9	3072
*B. abortus* 544	WGS	–	9	3120
*B. abortus* NCTC 8038	WGS	–	10	3044
*B. abortus* Tulya	WGS	3	10	3261

### Chromosome Arrangement

**FIGURE 6:**
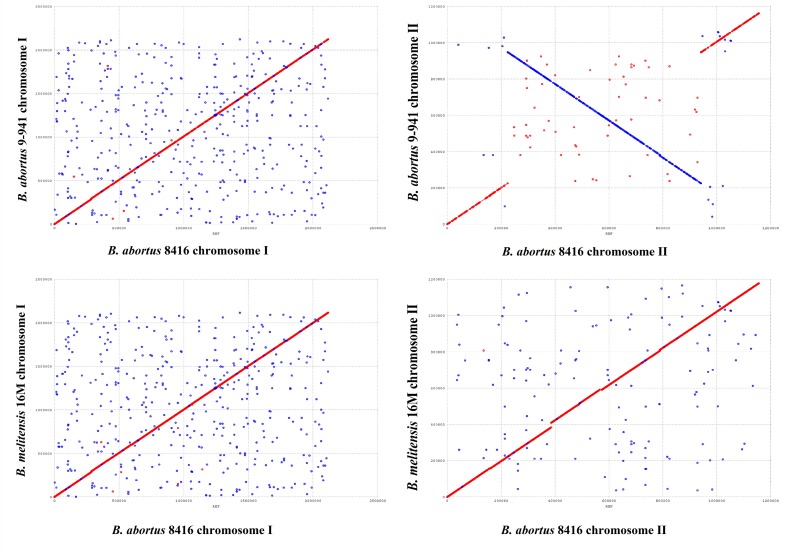
Genome arrangement of *Brucella abortus* strain Bab8416 small Chromosome. The four complete *B. abortus* genomes, including *B. abortus* Bab8416, were compared to *B. abortus* Bab9-941 and arrangements only appeared in small Chromosome of *B. abortus* Bab8416.

In comparison with Bab9-941, a large fragment (420 kb) re-arrangement in small chromosome of Bab8416 was found by using MUMmer (Figure 6). Re-arrangements in *Brucella* species have been previously reported (Sieira et al., 2000; Jiang et al., 2013a), however, this one proved to be exceptional. Compared with other *B. abortus* genomes observed here, the re-arrangement in Bab8416 was specific and displayed a closer linear relationship with *B. melitensis* 16M than the other *B. abortus* genomes. As mentioned above, Bab8416 shared the same phage typing status with *B. melitensis bv* 1 strain 16M; strongly similar genomic structures were also shown to exist between these two strains.

Nevertheless, neither IS elements nor tRNA operons usually responsible for genome re-arrangement were detected in the terminal region of the Bab8416 re-arrangement sequence. Three genes, *BMEII0292*, *BMEII0293*, and *BMEII1009*, were truncated or incomplete at the terminal fragment in other *B. abortus* strains. Both *BMEII0292* and *BMEII1009* contain a GGDEF domain that enables them to generate the cyclic di-GMP (c-di-GMP), a kind of secondary messenger central in regulating bacteria adaptive responses. In addition, analysis of protein-protein interactions using STRING database (Franceschini et al., 2013) indicated that *BMEII0293* encodes a hypothetical protein that is tightly associated with the synthesis and degradation of c-di-GMP. In *B. melitensis*, 11 c-di-GMP-metabolizing proteins had been inferred to regulate c-di-GMP metabolism (Petersen et al., 2011). The structure of these 11 genes were verified to be intact in Bab8416, but *BMEII0929, BMEII0292* and *BMEII1009*, were found absent in Bab941 and some *B. abortus* strains.

### Deletions and Insertions

Compared with SPS Bab9-941, 49 indels (≥20 bps), including 25 deletions and 24 insertions, were found in the Bab8416 genome (Tables 3, 4). A 16.5 kb region is absent from Bab8416 genome and a 3.2 kb region appears to be unique. Only four large regions (>500 bp) were represented by deletions, one Region of Differences (RD) 1 in chromosome I and three (RD2–RD4) in small chromosome. Genes lost in these regions are determined by referencing the annotation of *B. abortus* 9–941. The details of deletions and associated ORFs are shown in Table 3.

**TABLE 3:** ORFs related to deletions in *B. abortus* str.8416 compared to *B. abortus* 9–941 genome.

			Region	Associated	Gene	Gene length %	
BAB9-941 coordinate			length	genes	length	in RD region	Gene functions
Chr1	Deletion	80475..80812	338	BruAb1_0072	2,271	14.88%	Hypothetical protein
	Deletion	85269..85303	35	BruAb1_0075	750	4.82%	Amino acid efflux LysE family protein
	Deletion	88412..88434	23	BruAb1_0079	384	0.52%	Hypothetical protein
	RD1	287585..295517	7,933	BruAb1_0284	1,767	100.00%	Phage integrase family site specific recombinase
				BruAb1_0286	195	100.00%	Hypothetical protein
				BruAb1_0287	618	100.00%	Resolvase family site-specific recombinase
				BruAb1_0289	228	100.00%	Hypothetical protein
				BruAb1_0290	285	100.00%	Hypothetical protein
				BruAb1_0291	261	100.00%	Hypothetical protein
				BruAb1_0292	390	100.00%	Hypothetical protein
	Deletion	375984..376015	32	BruAb1_0371	1,128	2.84%	ABC transporter substrate-binding protein
	Deletion	1040055..1040246	193	BruAb1_1057	1,596	4.04%	DEAD/DEAH box helicase
	Deletion	1774700..1774731	32	BruAb1_1803	405	8.21%	30S ribosomal protein S16
	Deletion	1795037..1795098	62	BruAb1_1825	711	8.90%	Hypothetical protein
Chr2	Deletion	156432..156703	272	BruAb2_0168	5,952	17.87%	Outer membrane transporter
	RD2	158847..159637	791				
	RD3	376963..382403	4,088	BruAb2_0377	1,335	62.76%	FAD-binding oxidoreductase
				BruAb2_0378	420	100.00%	Hypothetical protein
				BruAb2_0379	1,011	100.00%	Epimerase
				BruAb2_0380	1,455	100.00%	Aminotransferase
	Deletion	620905..620941	37	BruAb2_0616	1,143	3.25%	Major facilitator family transporter
	RD4	701096..701938	843	BruAb2_0690	477	100.00%	IS711, transposase orfB
				BruAb2_0691	312	100.00%	Transposase orfA
	Deletion	711185..711248	64	BruAb2_0698	1,296	7.27%	Branched-chain alpha-keto acid dehydrogenase subunit E2

**TABLE 4:** ORFs in insertions in *B. abortus* 8416 compared to *B. abortus* 9-941 genome.

		Region	Associated	Gene	ORF length %	
BAB8416 coordinate		length	ORFs	length	in Insertions	Gene Function
Chr1	15707..15843	137	BAB8416_I0012	1155	11.86%	ABC transporter, substrate-binding protein
	374342..374383	42	BAB8416_I0362	2019	2.08%	Xanthine dehydrogenase, molybdenum binding subunit
	643653..643790	138	BAB8416_I0630	1104	12.50%	ATP/GTP-binding site motif A
	1035800..1037009	1210	BAB8416_I1044	651	100.00%	Diguanylate cyclase/phosphodiesterase domain
	1040674..1040705	32	BAB8416_I1049	480	6.67%	Multidrug resistance protein A
	1409719..1409750	32	BAB8416_I1431	195	16.41%	FIG00450652: hypothetical protein
Chr2	4629..4760	132	BAB8416_II0007	912	14.47%	Nucleoside ABC transporter, permease protein 2
	10010..10058	49	BAB8416_II0012	726	6.75%	4’-phosphopantetheinyl transferase entD
	231155..231181	27	BAB8416_II0234	570	4.74%	Nitric oxide reductase activation protein NorE
	248732..248754	23	BAB8416_II0253	828	2.78%	Various polyols ABC transporter, ATP-binding component
	459512..459556	45	BAB8416_II0463	1335	3.37%	Branched-chain alpha-keto acid dehydrogenase, E1 component, alpha subunit
	572539..572616	78	BAB8416_II0569	1203	6.48%	Acetyl-CoA acetyltransferase
	849446..849532	87	BAB8416_II0843	1107	7.86%	RND efflux membrane fusion protein
	942069..942905	837	BAB8416_II0941	1047	46.51%	Putative Heme-regulated two-component response regulator
			BAB8416_II0942	1233	14.27%	l-2-hydroxyglutarate oxidase

Eight genes, *BruAb1_0284-0292*, were located in RD1 region. *BruAb1_0284* and *BruAb1_0287* are specific recombinases, belonging to phage integrase and resolvase families, respectively. *BruAb1_0285* and *BruAb1_0288* were annotated as pseudo genes and the others were labeled hypothetical proteins. In addition, we further detected the RD1 region in 200 *B. melitensis* genomes and 197 *B. abortus* genomes by using BLASTn. In all of *B. melitensis* 200 strains we could not found any sequence similar with RD1. While 127 out of 197 *B. abortus* strains could be found the sequences with identity higher than 99% and coverage over 90% (Supplementary Table S4). These evidences above showed that RD1 was exclusively specific to *B. abortus* and the insert event should occur after the differentiation of the most recent common ancestor of *B. abortus* 9–941 and Bab8416. RD2 and another small deletion are involved in the locus of an outer membrane transporter, *BruAb2_0168*. An earlier study confirmed that this locus was conserved between *B. abortus* (Halling et al., 2005), but variation is present in Bab8416. RD3 contains four genes, *BruAb2_0377* to *BruAb2_0380*. *BruAb2_0377* encodes FAD-binding oxidoreductase. *BruAb2_0378* was defined as a hypothetical protein. *BruAb2_0379* encodes an epimerase that catalyzes the transformation of dTDP-glucose to dTDP-4-oxo-6-deoxy-D-glucose. *BruAb2_0380* encodes an amino transferase that participates in arginine and proline metabolism, metabolic pathways and biosynthesis of secondary metabolites. Two intact genes and one partial gene are encoded by RD4. The two complete genes, *BruAb2_0690* and *BruAb2_069*, encode transposase.

Inserted regions specific to Bab8416 are shown in Table 4. Among the 20 Bab8416 specific regions, six regions are located at intergenic spacer (IGS) and fifteen ORFs are involved in the other 14 insertions. All of these ORFs are annotated with known functions.

### Variant ORFs

The variant ORFs were identified by BLASTn method. The results are shown in Table 5. In consideration of the prediction discrepancy and the restriction of software, we searched these ORFs within these five genomes. BLASTn results showed that 144 Bab9-941 ORFs were found deleted or incomplete in Bab8416 and 129 Bab8416 ORFs were found to be Bab8416 specific. These deletions may be partly responsible for the unusual *Brucella* phage status of Bab8416.

**TABLE 5:** Classification of *B. abortus* strain Bab8416 specific SNPs associated ORFS.

Function class	ORF numbers
Amino acid transport and metabolism	5
Carbohydrate transport and metabolism	3
Cell envelope biogenesis, outer membrane	3
Cell motility and secretion	3
Coenzyme metabolism	2
Function unknown	1
General function prediction only	2
Inorganic ion transport and metabolism	7
Lipid metabolism	1
Nucleotide transport and metabolism	1
Transcription	3
Translation, ribosomal structure and biogenesis	5
Not in COGs	27

### SNPs

A total of 1,373 SNPs were identified between Bab8416 and Bab9-941. Using *B. abortus* 9–941 as a reference, 336 SNPs were intergenic and 1,036 SNPs were located in the ORFs. In addition, 518 genes-encoding proteins showed amino acid changes caused by 632 non-synonymous SNPs. As the SNP number was large, we inferred that these markers appeared in Bab8416 could be the characteristics of ST2. Since no other complete genomes of ST2 were available, we chose to utilize the existing draft genomes. In consideration of insuring the quality of sequencing and assembly, only the draft genomes with contig numbers less than 12 were selected. The MLST typing results of these genomes are shown in Supplementary Table S1. Fifteen out of 95 genomes were identified to be ST2. We tested the SNPs between the 16 ST2 genomes and found that overwhelming majority (95.05%) of former identified SNPs were verified to be potential markers of ST2 strains and only 68 SNPs appeared to be Bab8416 specific. The detailed SNP annotations are present in Supplementary Table S2 and the Bab8416 specific SNP involved genes are presented in Supplementary Table S3.

### LPS Synthesis

Lipopolysaccharide is tightly associated with the virulence of pathogens and the efficiency of corresponding vaccines. *Brucella* with rough lipopolysaccharide (R-LPS) was lysed by *Brucella* phage R/C, and is host specific (Hammerl et al., 2017). In *Brucella*, genes essential in synthesizing LPS and developing a smooth phenotype have been located at the Wbk region of chromosome I (Godfroid et al., 2000; Gonzalez et al., 2008; Zygmunt et al., 2009). Inactivation of formyltransferase (*wbkC*) gene is the significant factor that contributes to rough phenotype (Lacerda et al., 2010). BLASTn results showed that none of these genes were deleted/missing in Bab8416. Four non-synonymous mutations were identified in Bab8416 LPS genes, only one (*BruAb1_1699*) was found not belonging to ST2. This gene encodes an OmpA family protein, which is tightly related to flagellar protein production and also related to the efflux pump.

### Virulence Factors

Bab8416 was isolated from a patient with clinical brucellosis, indicating that this strain was virulent. The presence of 23 *Brucella* virulence factors confirmed by VFDB was tested in the Bab8416 isolate. Bab8416 was found to have a full complement of these loci. BLASTp results showed that eleven genes were 100% identical, eight genes had point mutations, and short deletions were found in the other four genes with only one deletion being present in *VFG2217.* In addition, compared to *BAB1_0069*, a putative outer membrane protein considered to be a virulence factor, a 133 amino acid deletion is present in this locus of Bab8416. We inferred these changes might exert some influence on the virulence of Bab8416 but not that much to cause high level attenuation as it is still a pathogenic bacterium.

## Conclusion

Combining NGS sequencing technology and comparative genomics analysis, the complete genome sequence of *B. abortus* SPR strain Bab8416 was obtained and specific genetic characteristics of *B. abortus* SPR were comprehensively investigated in this study. Study of smooth LPS related genes showed that Bab8416 does share same LPS key genes with other *B. abortus* SPR strains, which supported veracity of previous phenotype screening results. The gold standard for *Brucella* characterization is still based on specific properties of the bacteria. None of the available molecular typing methods covers all currently known species and biovars of the genus *Brucella* (Hammerl et al., 2017). The difference between biotyping and genotyping of some special strain need further analysis not only on genomic but protein expressive level, because the host strains co-evolute with their special phages. The importance of individual amino acids of the tail collar protein for the host range of the *Brucella* phages has not yet been investigated. To avoid diverging lysis patterns, examine the phage genomes by sequencing were recommended if the lysis results are inconsistent on the same indicator strains (Hammerl et al., 2017).

Bab8416 has a genetic profile different from that typically found in most *B. abortus* strains. The arrangement sequences in small chromosome resulted in the truncation of c-di-GMP synthesis. The indels within SPS and SPR *B. abortus* showed that two evolutionary branches might have diverged at a far phylogenetic node. Plentiful point mutations were identified to be Bab8416 specific while the majority of the point mutations were verified to be ST2 characteristic of *B. abortus*. While few Bab8416 SNPs were identified, SNPs might still exert a significant influence on phage typing status. Despite the unique genetic characteristics of Bab8416 uncovered in this study, full details of its resistance to phage have not yet been elucidated at the genomic level. Our findings established some novel molecular mechanisms underlying *Brucella* sensitivity to brucellapages that might contribute to improving our understanding on *Brucella* phenotyping.

## Author Contributions

X-ML and Y-XK performed genomic sequencing and comparative genomic analyses and wrote the manuscript. LL, E-HJ, and D-RP performed Brucella MLVA typing and phage typing. HJ, C-CZ, and JH performed Brucella MLST typing and PCR sequencing. Y-FC, X-KG, and YZ designed the whole experiments and revised the manuscript.

## Conflict of Interest Statement

The authors declare that the research was conducted in the absence of any commercial or financial relationships that could be construed as a potential conflict of interest.

## Footnotes

1 http://www.bacterio.net/brucella.html

2 http://mlva.u-psud.fr/brucella/
